# Shining a light on the evidence for hydroxychloroquine in SARS-CoV-2

**DOI:** 10.1186/s13054-020-02894-7

**Published:** 2020-04-28

**Authors:** Nicholas E. Ingraham, David Boulware, Matthew A. Sparks, Timothy Schacker, Bradley Benson, Jeffrey A. Sparks, Thomas Murray, John Connett, Jeffrey G. Chipman, Anthony Charles, Christopher J. Tignanelli

**Affiliations:** 1 Division of Pulmonary and Critical Care, Department of Medicine, University of Minnesota, MMC 195, 420 Delaware St SE, Minneapolis, MN 55455 USA; 2 Division of Infectious Diseases and International Medicine, Department of Medicine, University of Minnesota, Minneapolis, USA; 3 Division of Nephrology, Department of Medicine, Duke University, Durham, NC USA; 4 Division of Medicine and Infectious Disease, Department of Medicine, University of Minnesota, Minneapolis, MN USA; 5 Division of General Internal Medicine, Department of Medicine, University of Minnesota, Minneapolis, MN USA; 6 Department of Medicine, Brigham and Women’s Hospital; Harvard Medical School, Division of Rheumatology, Inflammation, and Immunity, Boston, MA USA; 7 Division of Biostatistics, School of Public Health, University of Minnesota, Minneapolis, USA; 8 Division of Acute Care Surgery, Department of Surgery, University of Minnesota, Minneapolis, MN USA; 9 Department of Surgery, University of North Carolina School of Medicine, Chapel Hill, NC USA; 10 School of Public Health, University of North Carolina School of Medicine, Chapel Hill, NC USA; 11 Institute for Health Informatics, University of Minnesota, Minneapolis, MN USA

**Keywords:** Evidence-based medicine, COVID-19, Hydroxychloroquine, Pandemic, RCT, SARS

## Background

The 2020 COVID-19 pandemic has stunned the world, financial markets, and healthcare systems. Researchers are rushing to identify effective treatments while maintaining rigorous adherence to the scientific method. Clinicians are doing their best to provide evidence-based care in a setting of very little good evidence. To date, no effective treatments exist for COVID-19 management. Unfortunately, traditional and social media coupled with world leader commentary have led some to believe hydroxychloroquine offers a bona fide cure and even prevention. The purpose of this commentary is to review the medical literature related to hydroxychloroquine building on knowledge over the past 17 years since the 2003 SARS-CoV epidemic.

## Main text

### Pre-clinical evidence

Hydroxychloroquine has been used for the treatment of malaria since 1955 and is approved for the treatment of rheumatoid arthritis and lupus. Currently, the potential mechanism of action for hydroxychloroquine’s effect on SARS-CoV1 and SARS-CoV2 is not fully known. It is hypothesized that increases in endosomal pH may inhibit viral fusion and replication with interference in ACE2 receptor glycosylation or Sigma-1 receptor [1, 2]. Chloroquine and hydroxychloroquine seem effective in killing SARS-CoV in vitro [1, 3]. Recent reports show it also may be effective at killing SARS-CoV-2-infected cells in vitro [4]. Unfortunately, effective treatments in vitro frequently do not translate in vivo and the efficacy of hydroxychloroquine is yet to be determined.

Pre-clinical animal (in vivo*)* models more accurately model human safety and efficacy and enable better prediction of translational failure into humans. Chloroquine was not found to be efficacious in mouse models infected with SARS-CoV despite multiple dosing schedules and treatment routes [5]. To date, no pre-clinical studies have evaluated the efficacy of hydroxychloroquine in the current SARS-CoV-2 pandemic.

### Current evidence

A recent article published in Chinese found no benefit with chloroquine in a 1:1 randomized trial with 30 patients [6]. As yet there are no published randomized controlled trials of hydroxycholoroquine in SARS-CoV-1 or 2. Recently, a publication by Gautret et al. has been touted by non-medical public figures as proof of a cure for COVID-19 [7]. This has led to significant interest in news outlets, social media, and the general public. This study was a non-randomized, non-blinded, open label, and underpowered trial. Given these limitations, it does not meet the rigor for evaluation of scientific efficacy. To illustrate, Gautret et al. treated 26 patients with hydroxychloroquine (six received concomitant azithromycin) that met study inclusion criteria. The control group was subsequently made up of 16 patients who did not meet the study inclusion criteria. The primary outcome was viral load, defined by a cycle time (CT) threshold of 35. CT is the number of reverse transcription PCR cycles necessary to detect the presence of viral RNA. A lower CT is associated with increased viral load. A CT greater than or equal to 35 was deemed as a negative viral titer. It was unclear whether this threshold was set a priori or post hoc. After enrollment of 42 patients, an interim analysis was conducted and published. A critical result was the exclusion of six patients from the treatment arm. One died, three decompensated requiring transfer to the ICU, one withdrew from the study due to drug-related complications, and one was lost to follow-up. These patients were excluded by the authors from final analysis. The authors then compared viral titers from 20 patients that received hydroxychloroquine (or combination with azithromycin) with the 16 control patients that were ineligible to receive hydroxychloroquine. In unadjusted analyses, the authors identified significantly reduced viral titers in the hydroxychloroquine arm. No comment was made regarding the higher mortality, complication, and adverse event rate in the hydroxychloroquine group.

Excluding those who did poorly with hydroxychloroquine is a biased analysis that impacts the potential validity of the study. Reincorporation of the 6 patients into the statistical analysis would have significantly changed the results of this study. In the hydroxychloroquine group, 5 of 26 (19.2%) of COVID-19 patients suffered death, medical deterioration, or adverse event compared with 0 (0%) in the control arm (Barnard’s test: *p* = 0.07) with a number needed to harm (NNH) of 5.2.

## Conclusion

Until data from randomized controlled trials are available, we suggest caution utilizing hydroxychloroquine off label for patients with COVID-19. Reports of overdoses are now occurring. There are currently no evidence supporting hydroxychloroquine as prophylaxis, but unfortunately these data are being extrapolated to the indication potentially resulting in drug shortages for patients with rheumatic diseases who require this medication. Furthermore, we caution medical and world leaders against premature comments of treatment efficacy during the COVID-19 pandemic. Such comments may exacerbate shortages for patients reliant on these medications or cause harm due to medication side effects and overdose [8].

## Data Availability

Not applicable.
